# Feasibility of a simultaneously integrated boost concept for hypofractionated stereotactic radiotherapy of unresected brain metastases

**DOI:** 10.1186/s13014-023-02266-9

**Published:** 2023-05-22

**Authors:** Christine Kornhuber, Stephan Ensminger, Patrick Hübsch, Martin Janich, Chris Andre Leucht, Dirk Vordermark, Christian T. Dietzel

**Affiliations:** Department of Radiation Oncology, Martin Luther University Halle-Wittenberg, Ernst-Grube-Str. 40, 06120 Halle (Saale), Germany

**Keywords:** Brain metastases, Hypofractionated stereotactic radiotherapy, Simultaneously integrated boost

## Abstract

**Background:**

In stereotactic radiotherapy, dose is prescribed to an isodose surrounding the planning target volume (PTV). However, the desired dose inhomogeneity inside the PTV leaves the specific dose distribution to the gross tumor volume (GTV) unspecified. A simultaneously integrated boost (SIB) to the GTV could solve this shortcoming. In a retrospective planning study with 20 unresected brain metastases, a SIB approach was tested against the classical prescription.

**Methods:**

For all metastases, the GTV was isotropically enlarged by 3 mm to a PTV. Two plans were generated, one according to the classical 80% concept with 5 times 7 Gy prescribed (on D_2%_) to the 80% PTV surrounding isodose (with D_98%_(PTV) ≥ 35 Gy), and the other one following a SIB concept with 5 times 8.5 Gy average GTV dose and with D_98%_(PTV) ≥ 35 Gy as additional requirement. Plan pairs were compared in terms of homogeneity inside GTV, high dose in PTV rim around GTV, and dose conformity and gradients around PTV using Wilcoxon matched pairs signed rank test.

**Results:**

The SIB concept was superior to the classical 80% concept concerning dose homogeneity inside GTV: Heterogeneity index of GTV was in the SIB concept (median 0.0513, range 0.0397–0.0757) significantly (p = 0.001) lower than in the 80% concept (median 0.0894, range 0.0447–0.1872). Dose gradients around PTV were not inferior. The other examined measures were comparable.

**Conclusion:**

Our stereotactic SIB concept better defines the dose distribution inside PTV and can be considered for clinical use.

## Background

Stereotactic radiotherapy (SRT) is a cornerstone in the modern era of treating brain metastases. It was originally developed for the precise ablation of a single or limited number of lesions ineligible for surgical resection, e.g., due to their localization or perioperative risk profile of the patient.

Single-fraction schedules, i.e., stereotactic radiosurgery (SRS), have resulted in promising 1-year local control rates of about 80% [1]. As preservation of neurocognitive function has become a more important outcome factor for patients with multiple brain metastases, SRS has been shown to be a suitable alternative—in patients with up to ten brain metastases—to whole-brain radiotherapy (WBRT) regarding toxicity and local control [2]. SRS may even result in an improved sparing of structures important for memory consolidation like the hippocampus [3].

On the downside, there is a non-negligible risk of radionecrosis (RN) as an intermediate to late effect of treatment after SRS. As the risk of RN increases with the size of a lesion, deescalating dose levels per tumor volume have been developed [4, 5]. Unfortunately, there is evidence that this dose de-escalation leads to an impaired local control rate [6]. Hypofractionated schedules with a small number of sessions seem to be a viable option to better exploit the therapeutic ratio between local control and toxicity in larger brain metastasis [7, 8].

Very recently, Tanenbaum et al. reported that the risk of RN after hypofractionated stereotactic radiotherapy (HFSRT) with five fractions was significantly associated with the presence of hot spots within the expansion margin from clinical target volume to PTV [9]. This suggests a clinical need for additionally controlling the dose distribution inside the target volume instead of only looking upon dose conformity and gradients outside of it as in classical stereotactic prescribing concepts.

In stereotactic radiotherapy, dose is prescribed to the outer border of the PTV or to an isodose surface that most optimally conforms to it [10]. To achieve good target conformity and sufficiently steep dose gradients to the surrounding normal tissue, dose inhomogeneity within the PTV is accepted or even desired. The level of inhomogeneity, defined by near minimum and near maximum dose in the PTV, is commonly included in the prescription, but the exact dose distribution within the PTV is not specified. Mean dose to the GTV, for example, can vary, depending on treatment technique, target size and planning person. To overcome this lack of plan comparability, Wilke et al. showed in a multi-institutional planning study for lung lesions that a multiparameter prescription with normalization to the mean dose in the internal target volume (ITV) combined with further objectives for PTV and ITV achieves consistent and reproducible dose distributions [11].

In our institution, single brain metastases are often treated using 5 fractions of 7 Gy prescribed to the 80% isodose closely surrounding the PTV: D_98%_(PTV) ≥ 35 Gy; D_2%_(PTV) = 43.75 Gy. Like Wilke et al. [11], we considered a multiparametric concept based on normalization to the mean dose in the GTV. We wanted to guarantee a specific dose to the GTV whatever its size with a better homogeneity.

The new concept was designed as a simultaneously integrated boost (SIB) for the GTV, where the boundary condition of our traditional 80% concept (D_98%_(PTV) ≥ 35 Gy) was maintained and the GTV mean dose level was arbitrarily set to 3% lower than the near maximum dose (43.75 Gy) of the traditional concept. The 3% reduction served as a precaution to prevent the new concept from being significantly "hotter" than the old one in larger lesions.

$${\text{New}}{\mkern 1mu} {\text{SIB}}{\mkern 1mu} {\text{concept}}:5{\mkern 1mu} \times {\mkern 1mu} 8.5Gy{\mkern 1mu} = {\mkern 1mu} 42.5Gy\,as{\mkern 1mu} D_{{mean}} \left( {GTV} \right);{\mkern 1mu} D_{{98\% }} \left( {PTV} \right){\mkern 1mu} \ge {\mkern 1mu} 35Gy.$$

$$80\% \, concept:5\, \times \,7 Gy\, = \,35 Gy\, as \, 80\% \, of \, D_{2\% } (PTV).$$

The difference between the two concepts can be illustrated as follows: In the 80% concept, any dose profile through PTV and GTV will look like a hill rising from 80% of the prescription dose at the PTV edge (35 Gy) to 100% at the dose summit (43.75 Gy). The summit can have any position and shape and can even be located at the GTV edge in case of poor planning. In the SIB concept, however, the same profile resembles a mesa, again with 35 Gy at the PTV edge but with an upper level of 42.5 Gy extending across the entire width of the GTV. This means that in the SIB concept, the GTV is ideally surrounded by the 40.38 Gy isodose (95% of the plateau dose level) and its mean dose is 42.5 Gy, while in the 80% concept, the minimum specified isodose surrounding the GTV is only 35 Gy and its mean dose is unspecified.

In a single-institutional planning study with 4 experienced planners, we tested the new SIB concept against our traditional 80% concept with a series of different-sized lesions to see if it can homogenize the dose distribution inside the GTV, without inducing hot spots in the area between GTV and PTV border and while maintaining conformity to the PTV and steep dose gradients around it.

## Methods

### Selection of lesions, immobilization, imaging, and contouring

20 brain metastases out of 17 patients recently treated in our institution were chosen for the study. Lesions were selected to cover the full range of tumor sizes defined in protocol 90–05 of the Radiation Therapy Oncology Group (RTOG). No critical structures were in close proximity. Immobilized in a stereotactic mask (Brainlab AG, Feldkirchen, Germany) the patients had been scanned with a computerized tomography slice thickness of 1.25 mm. In the treatment planning system (Raystation9b, RaySearch Laboratories AB, Stockholm, Sweden), GTV and relevant organs at risk including whole brain were contoured based on a co-registered T1-weighted contrast-enhanced magnetic resonance image series. PTV was constructed from GTV by adding an isotropic margin of 3 mm. The margin is still within the recommendations of the national stereotactic radiotherapy working group [12]. It is necessary despite the use of a stereotactic mask and daily image guidance because of geometric inaccuracies of our imaging system (Elekta XVI cone beam computerized tomography) and the treatment device (Synergy, Elekta Solutions AB, Stockholm, Sweden) with 5 mm leaf width in isocenter distance. PTV sizes ranged from 1.4 to 29.5 cm^3^ (median 7.4 cm^3^) corresponding to equivalent effective diameters of isovolumetric spheres from 1.4 to 3.8 cm (median 2.4 cm).

### Treatment planning and dose calculation

Treatment planning was performed on anonymized patient data. Four experienced planners were asked to prepare a plan for 10 lesions each, following the guidelines in Table 1. To avoid bias, each planner only worked according to either the 80% concept or the SIB concept, so that in the end there were two plans from two different planners for each lesion. Care was taken to randomly assign the lesions such that each of the four possible combinations of planners occurred an equal number of times. In the planning system, per lesion two separate cases had been created for the two concepts. Additionally, the two planners assigned to the 80% concept were not familiar with the SIB concept. To reflect clinical practice, treatment technique was left to the planners’ own choice. With inverse planning, however, effort was to be made to mimic a dose distribution that is characteristic of 3D conformal radiotherapy. In no case, the dose maximum was to lie outside the GTV. Dose was calculated with collapsed cone algorithm in a 1 mm (isotropic) dose grid. A recalculation with a Monte Carlo dose engine was not necessary, because the Monte Carlo algorithm does not yield different results in the soft tissues of the brain. As this was a planning study, we did not perform dosimetric verification of inverse modulated plans as is normally done with Octavius 4D combined with Detector 1600 SRS (PTW Freiburg, Germany).

**Table 1:** Details of planning guidelines for 80% and SiB concept

80% concept	SIB concept
5 × 7 Gy = 35 Gy as 80% on D_2_(PTV)	5 × 8.5 Gy = 42.5 Gy on D_mean_(GTV)
36 Gy > D_98_(PTV) ≥ 35 Gy	36 Gy > D_98_(PTV) ≥ 35 Gy
D_max_(PTV) = D_max_(GTV) = 45 Gy	D_max_(PTV) = D_max_(GTV) = 44 Gy

### Data collection, processing, and evaluation

The following data were collected from each plan: Technical information (treatment technique, beam quality, number of arcs, number of monitor units and segments), dose-volume histogram parameters of PTV (D_98%_, D_50%_, D_2%_, D_max_, V_35Gy_), GTV (D_98%_, D_mean_, D_2%_, D_max_), PTV without GTV (D_2%_), and whole brain (V_24Gy_ as a predictor for RN). For the GTV, a heterogeneity index HI was calculated as

1 $${\varvec{H}}{\varvec{I}}(GTV)=\frac{{{\varvec{D}}}_{2\boldsymbol{\%}}-{{\varvec{D}}}_{98\boldsymbol{\%}}}{{{\varvec{D}}}_{50\boldsymbol{\%}}}(GTV)$$

Furthermore, as recommended by Wilke et al. [10], a high dose conformity index according to Paddick, CI_Paddick_, was assessed for the PTV

2 $${{\varvec{C}}{\varvec{I}}}_{{\varvec{P}}{\varvec{a}}{\varvec{d}}{\varvec{d}}{\varvec{i}}{\varvec{c}}{\varvec{k}}}=\frac{{\left({{\varvec{V}}}_{35{\varvec{G}}{\varvec{y}}}\left(PTV\right)\right)}^{2}}{{{\varvec{V}}}_{35{\varvec{G}}{\varvec{y}}}\cdot {\boldsymbol{ }{\varvec{V}}}_{{\varvec{P}}{\varvec{T}}{\varvec{V}}}}$$

V_35Gy_ (PTV), V_35Gy_ and V_PTV_ denote the volume of the PTV inside the 35 Gy-isodose, the complete volume inside this isodose and the volume of the PTV, respectively. As a measure for the dose fall-off outside the PTV, we assessed the spatially averaged dose gradient SADG^*^ proposed by Wösle in 2018 [13]. SADG^*^ is the spatially averaged dose difference quotient in radial direction between isodose surfaces D_1_ and D_2_. Its unit is Gy/mm or %/mm.

3 $${{\varvec{S}}{\varvec{A}}{\varvec{D}}{\varvec{G}}}^{\boldsymbol{*}}{|}_{{{\varvec{D}}}_{2}}^{{{\varvec{D}}}_{1}}=\frac{{{\varvec{D}}}_{2}-{{\varvec{D}}}_{1}}{{\left({{\varvec{r}}}_{2}-{{\varvec{r}}}_{1}\right)}_{{\varvec{a}}{\varvec{v}}{\varvec{g}}}}= \frac{{{\varvec{D}}}_{2}-{{\varvec{D}}}_{1}}{{\Delta r}_{\Delta D}}<0$$

In [14], a simple method for its determination is provided: The PTV is modelled as an ellipsoid, and two concentric, equidistant ellipsoids are fitted to the isodose surfaces D_1_ and D_2_ by Newton’s iteration method to obtain Δr_ΔD_. This algorithm was implemented by one of us into the planning system by means of a script. For D_1_ the PTV-enclosing isodose value 35 Gy and for D_2_ half of it, 17.5 Gy, were chosen.

As a null hypothesis, we assumed that the two concepts, 80% and SiB, were equal. To judge the differences of the plan pairs, the Wilcoxon matched pairs signed rank test (two-sided) was employed [15] using Statistica (StatSoft, Tulsa OK, US). As V_24Gy_(brain) strongly depends on PTV size, a trend line was calculated first, and the differences of V_24Gy_(brain) between the two concepts were normalized to this trend line before applying the test.

## Results

### Technical parameters

Except from one minor deviation (D_98%_(PTV) = 34.9 Gy), all 40 plans fulfilled the requirements in Table 1 and were all included in the evaluation. They were designed either in volumetric modulated arc technique (VMAT—30 plans) or in dynamic conformal arc (DCA—10 plans) technique (7 DCA plans in the SIB concept and 3 in the 80% concept). The beam energy was always 6 MV, mostly flattening filter free; in the 80% concept, 12 plans (60%) utilized flattened beams. The SIB plans mostly consisted of more arcs; modal value was 5 versus 4 in the 80%-concept. The number of segments was slightly higher in the 80% concept (median 179, range 96–274) versus median 162, range 106 to 205 in the SIB concept, indicating that the SIB plans used shorter arcs.

### Statistical analysis

In Table 2 the results of the statistical analysis are summarized.

**Table 2:** Comparison of plan parameter statistics in the competing concepts, 80% and SiB

Parameter/median (range)	80% concept	SiB concept	p	Figure
Number of monitor units	1036 (904.4 to 1259)	933.7 (893.5 to 1221)	0.001	1a, 1b
number of segments	179 (96 to 274)	162 (106 to 205)	–	1b
D_mean_(GTV)/Gy	42.4 (41.0 to 43.3)	42.5	–	2a
D_98_(GTV)/Gy	40.1 (36.4 to 42.0)	41.1 (40.4 to 41.5)	0.01	2b
D_2_(GTV)/ Gy	43.9 (43.8 to 44.1)	43.4 (43.0 to 43.7)	–	2c
HI(GTV) = (D_2_−D_98_)/D_50_	0.0894 (0.0447 to 0.1872)	0.0513 (0.0397 to 0.0757)	0.001	2d
D_2_(GTV without PTV)/Gy	41.9 (41.1 to 43.2)	42.2 (41.3 to 42.8)	> 0.05	3a
CI_Paddick_(PTV)	0.88 (0.61 to 0.95)	0.89 (0.73 to 0.93)	> 0.05	3b
SADG*/Gy/mm	− 2.45 (− 4.16 to − 1.85)	− 3.14 (− 4.56 to − 2.02)	0.01	3c
V_24Gy_(brain) normalized [Values were created by normalizing original data to trend line in Fig. 3d]	1.10 (0.749 to 1.28)	0.968 (0.727 to 1.21)	0.001	3d

p is the error probability resulting from the Wilcoxon-Signed-Rank-Test when assuming a difference between the concepts. Missing p values indicate that the analysis of differences was not considered meaningful. In case of p values exceeding 0.05 there is no statistical evidence for a difference between the concepts

#### Number of monitor units

**Fig. 1:**
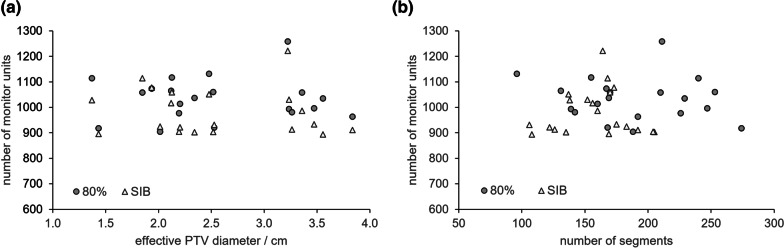
Plan monitor units versus effective PTV diameter (**a**) and versus number of beam segments (**b**) for the 80% and the SIB concept

The number of monitor units was independent of PTV size (Fig. 1a). On median, the 80% plans required 1036MU (range 904–1259MU) versus 933MU (range 893–1221MU) for the SIB plans. There was statistical evidence (p = 0.001) that the two concepts are different: On median, the SIB plans could manage with 10% less monitor units. This result can not be explained by the lower number of segments in the SIB concept: Fig. 1b clearly shows that the number of monitor units is independent of the number of segments in both concepts. Instead, it does indicate that the SIB plans are at least of equal if not of higher quality than the 80% plans.

#### Homogeneity inside GTV

**Fig. 2:**
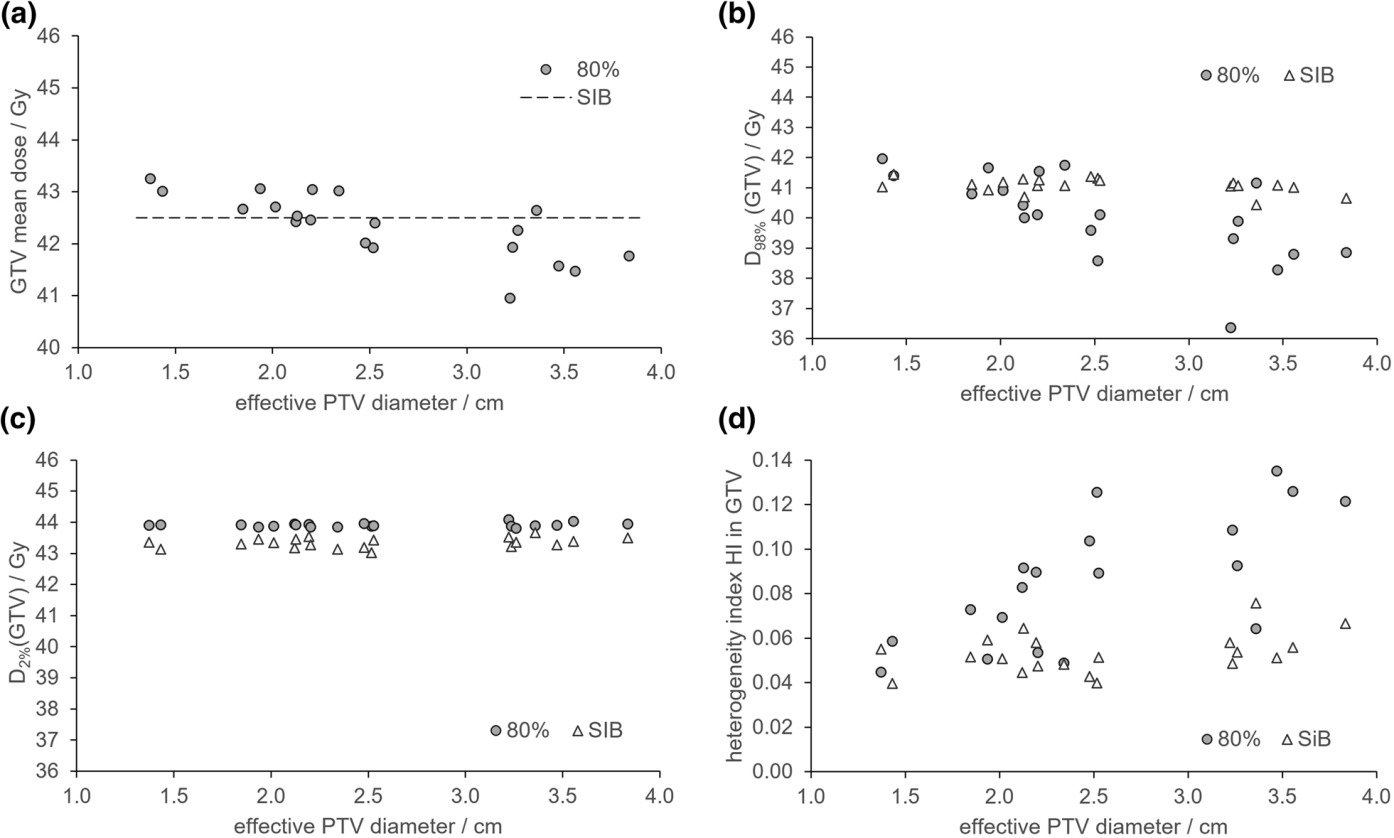
GTV mean dose (**a**), D_98%_ (**b**), D_2%_ (**c**), and heterogeneity index (**d**) versus effective PTV diameter for the 80% and the SIB concept

Mean dose D_mean_ in the GTV was by prescription 42.5 Gy in the SIB concept. In the 80% concept, it was on median not different (42.4 Gy) but ranged from 41.0 to 43.3 Gy (5%). A clear trend to lower values with increasing PTV sizes is visible (Fig. 2a), possibly due to the missing guidelines for GTV dose level. This same trend appeared still more pronounced when looking at GTV near minimum dose D_98%_ (Fig. 2b): It was significantly higher (p = 0.01) in the SIB concept (median 41.1 Gy, range 40.4–41.5 Gy) than in the 80% concept (median 40.1 Gy, range 36.4–42.0 Gy).

On the other hand, due to the more stringent limitation of GTV maximum dose, the near maximum dose D_2%_(GTV) was in the SIB concept lower than in the 80% concept (Fig. 2c). As a result, the heterogeneity index HI (Eq. 1) in the GTV (Fig. 2d) was in the SIB concept (median 0.0513, range 0.0397–0.0757) significantly (p = 0.001) lower than in the 80% concept (median 0.0894, range 0.0447–0.1872). The SIB concept therefore renders the GTV dose distribution more homogeneous and thus more reproducible.

#### High dose at PTV rim

**Fig. 3:**
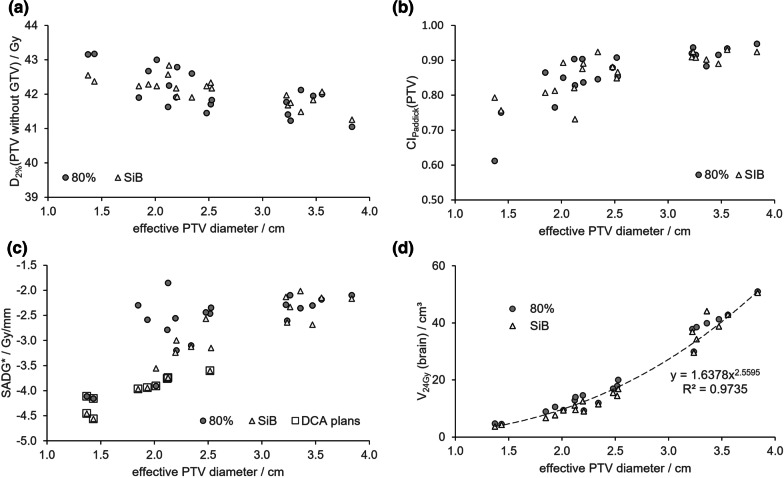
D_2%_ in ring area around GTV (**a**), Paddick high dose conformity index according to Eq. 2 (**b**), SADG* according to Eq. 3 (**c**), and V_24Gy_(brain) (**d**) versus effective PTV diameter for the 80% and the SIB concept. In c the plans in DCA technique are outlined by squares

Flattening the dose distribution inside the GTV poses the risk of pushing excessive dose into the marginal area between GTV and PTV border. The near maximum dose in this area, D_2%_ (PTV without GTV), however, did not differ in the two concepts (Fig. 3a).

#### Dose conformity

Regarding high dose conformity around PTV according to Eq. 2, there was also no significant difference between the concepts: CI_Paddick_ increased with increasing PTV size, a well-known and disadvantageous property of the index, and the differences in CI_Paddick_ between the two concepts grew smaller and smaller (Fig. 3b) in line with the fact that the relative leaf width decreased and the VMAT technique became more prevalent.

#### Dose gradient outside PTV

The mean dose fall-off outside the PTV, represented by the spatially averaged dose gradient SADG* according to Eq. 3 (Fig. 3c), was significantly (p = 0.01) steeper in the SIB concept. Figure 3c also reveals that the plans in dynamic conformal arc technique are in both concepts those with the largest dose gradients.

#### Integral brain dose

Steeper dose gradients around the PTV should result in lower integral brain dose. Brain volume V_24Gy_ with a minimum dose of 24 Gy (Fig. 3d) serving as a predictor for RN in an irradiation schedule with five fractions [16] was actually significantly (p = 0.001) lower in the SIB concept (median 13.5cm^3^, range 3.7–50.6 cm^3^) than in the 80% concept (median 15.8 cm^3^, range 4.6–51.0 cm^3^).

## Discussion

### Clinical relevance

Our planning study for HFSRT with 20 single brain metastases demonstrated that a multiparametric prescription (SIB concept) based on the mean dose in the GTV additionally to a PTV surrounding isodose is feasible for a wide range of PTV diameters (1.4–3.8 cm). Compared to our traditional (80%) concept with prescription to a PTV surrounding isodose as 80% of the normalization dose D_2%_ (PTV), the new SIB concept directly shapes the dose distribution inside the GTV and thus is expected to be better correlated to treatment outcome. The SIB plans were of good, if not even of better quality than the traditional plans. The study further showed that in the SIB concept, the improved homogeneity in the GTV was not accompanied by an increased near maximum dose in the surrounding marginal area of the PTV.

### Dose conformity and gradients outside PTV

Concerning high dose conformity to the PTV, the SIB concept was not inferior to the 80% concept. Within the 3 mm margin from GTV to PTV border the dose can fall easily to the required value without compromising GTV coverage. With respect to the average dose gradients outside the PTV, the SIB plans were even superior. Admittedly, this might be partly due to the higher number of DCA plans in the SIB concept (7 vs. 3), as also suggested by the findings of Brun et al. in a similar plan comparison [17]. Also, the beam quality (6MV flattened versus unflattened) could have had an influence on the dose gradients. Pokhrel et al. found in SBRT of lung lesions higher dose gradients outside the target border with VMAT plans using unflattened rather than flattened beams [18]. In the 80% concept, unflattened beams were applied in only 8 out of 20 plans, whereas in the SIB concept only the unflattened beam quality was utilized. Finally, especially with inverse optimization, the planner’s specific ambition to maximize dose gradients is of utmost influence on the result (see also Limitation of study section). For these reasons, it is not justified to attribute the steeper dose gradients found in SIB plans to that concept as such. Instead, it can be resumed that concerning dose gradients, the SIB concept is not inferior to the 80% concept.

### Integral brain dose and RN

Recent trials examining the likeliness of RN following HFSRT with a five-fraction dose design reported a moderate risk of around 5–9% [8, 19]. To keep it below 10%, Milano et al. recommended limiting the brain volume exposed to at least 24 Gy (V_24Gy_) to no more than 20 cm^3^ [16]. In our study, this threshold was exceeded for PTV diameters above 2.5 cm (Fig. 3d), regardless of the underlying dose concept. This might be a hint, that for large metastases a dose concept with five fractions is not a “first choice” in terms of toxicity. Ongoing studies investigate the role of further fractionation with treatments schedules of around 10–12 × 4 Gy [20]. A promising approach, however, might also be a concept like 8 × 5 Gy (80%), which is isoeffective to 5 × 7 Gy (80%) according to the LQ-model with α/β = 10 Gy. For this alternative concept, the following SiB concept can likewise be set up, and the results presented so far remain valid: *8* × *6 Gy* = *48 Gy as D*_*mean*_* (GTV) with D*_*98%*_* (PTV)* ≥ *40 Gy.*

### Limitation of study

Our study was not designed to clearly distinguish between concepts in terms of dose gradients around the PTV. For this, we would have had to prescribe the planning technique more rigorously or significantly increase the number of lesions and planning persons. It is quite possible that due to the better GTV coverage and the resulting steeper dose fall-off in the PTV rim, the SIB concept actually produces better dose gradients, but this would need to be investigated in a further study.

### New methodology

To our knowledge, this is the first planning study that systematically assessed the new gradient index SADG* proposed by Wösle in 2018. It is a one-dimensional approximation of the general two-dimensional anisotropic dose gradient SADG [14] and can quite easily be calculated. Contrary to other gradient measures like the gradient index GI by Paddick [21] which is in fact an isodose volume ratio, SADG* is defined as a dose gradient and as such shows a physically meaningful behavior with varying PTV sizes [14].

## Conclusion

Our novel multiparametric prescription concept (SIB) for hypofractionated stereotactic radiotherapy of brain metastases, based on the mean dose in the GTV and additionally on a PTV surrounding isodose, proved to be feasible for effective PTV diameters from 1.4 to 3.8 cm. In terms of high dose conformity and compactness of dose around the PTV, it was not inferior to our old 80% concept, which is only based on a PTV surrounding isodose as 80% of the normalization dose D_2%_. In contrast, the SIB concept was clearly superior concerning GTV homogeneity, which was not accompanied by excessive dose in the marginal area of the PTV around the GTV. The SIB concept can hence be considered for clinical use. It is suited to contribute to the standardization of planning in stereotactic radiotherapy.

## Data Availability

The datasets used and/or analysed during the current study are available from the corresponding author on reasonable request.

## Abbreviations

DCA: Dynamic conformal arc
FFF: Flattening filter free
GTV: Gross tumor volume
HFSRT: Hypofractionated stereotactic radiotherapy
ITV: Internal target volume
PTV: Planning target volume
RN: Radionecrosis
RTOG: Radiation Therapy Oncology Group
SADG*: Spatially averaged dose gradient
SIB: Simultaneously integrated boost
SRS: Stereotactic radiosurgery
SRT: Stereotactic radiotherapy
VMAT: Volumetric modulated arc therapy
WBRT: Whole-brain-radiotherapy
